# Supplementary material for: Changing POU dimerization preferences converts Oct6 into a pluripotency inducer
Source: EMBO Rep. 2016 Dec 22;18(2):319–33. doi: 10.15252/embr.201642958 (PMC5286379; doi:10.15252/embr.201642958)
Supplement: Supplementary file 1 — Appendix [file EMBR-18-319-s001.pdf]

APPENDIX

TABLE OF CONTENTS

Information	Page
Appendix Supplementary **Table S1**<br>**EMSA OLIGONUCLEOTIDES**	2
Appendix Supplementary **Table S2**<br>**ENERGY MINIMIZATION PROCEDURE**	3
Appendix Supplementary **Table S3**<br>**EQUILIBRATION PROCEDURE FOR MOLECULAR DYNAMICS SIMULATIONS**	4
Appendix Supplementary **Table S4**<br>**SEQUENCES OF OLIGONUCLEOTIDES FOR CLONING AND SITE-DIRECTED MUTAGENESIS**	5
Appendix Supplementary **Table S5**<br>**SEQUENCES OF PRIMERS FOR GENOTYPING OR qRT-PCR**	6
Appendix **Figure Legends**	7
Appendix **Fig. S1**	8
Appendix **Fig. S2**	9

APPENDIX SUPPLEMENTARY TABLE S1:

EMSA OLIGONUCLEOTIDES

DNA element	Sequence
MORE **(*OctOct*)**	TCCTCATGAATATTCATGAGGA
SoxOct	CCATGGACATTGTAATGCAAAAGAAGCTG

APPENDIX SUPPLEMENTARY TABLE S2:

ENERGY MINIMIZATION PROCEDURE

Step (no. of minimization steps, no. of steepest descent, no. of conjugate gradient steps)	Positional restrains<br>k_1 = force contstant	Distance restraints<br>(to preserve hydrogen bonds# and hydrophobic## interactions)<br>k_2 = force contstant
1 (5000, 100, 4900)	protein and DNA heavy atoms<br>k_1 = 25 kcal/mol · Å^2	-
2 (5000, 100, 4900)	protein and DNA heavy atoms<br>k_1 = 10 kcal/mol · Å^2	-
3 (5000, 100, 4900)	protein and DNA heavy atoms<br>k_1 = 5 kcal/mol · Å^2	-
4 (5000, 100, 4900)	protein and DNA backbone<br>k_1 = 5 kcal/mol · Å^2	Watson Crick base pairs and protein-DNA (sidechain-base) interactions<br>k_2 = 20 kcal/mol · Å^2
5 (5000, 100, 4900)	protein and DNA backbone<br>k_1 = 1 kcal/mol · Å^2	k_2 = 20 kcal/mol · Å^2
6 (5000, 100, 4900)	protein and DNA backbone<br>k_1 = 0.5 kcal/mol · Å^2	k_2 = 20 kcal/mol · Å^2
7 (5000, 100, 4900)	protein and DNA backbone<br>k_1 = 0.1 kcal/mol · Å^2	k_2 = 20 kcal/mol · Å^2
8 (5000, 100, 4900)	protein and DNA backbone<br>k_1 = 0.05 kcal/mol · Å^2	k_2 = 20 kcal/mol · Å^2
9 (5000, 100, 4900)	protein and DNA backbone<br>k_1 = 0.01 kcal/mol · Å^2	k_2 = 20 kcal/mol · Å^2
10 (5000, 100, 4900)	-	k_2 = 20 kcal/mol · Å^2
11 (5000, 100, 4900)	-	-

for hydrogen bonds, the donor acceptor distance threshold was 3.0

for hydrophobic interactions the distance threshold was 4.0 Å

APPENDIX SUPPLEMENTARY TABLE S3:

EQUILIBRATION PROCEDURE FOR MOLECULAR DYNAMICS SIMULATIONS

Step (time, ensemble, timestep)	T, Langevin damping coefficient P, Nose Hoover Langevin piston period and decay	Positional restrains k_1 = force contstant	Distance restraints (to preserve hydrogen bonds# and hydrophobic## interactions) k_2 = force contstant
1 (150 ps, NVT, 1 fs)	20-300 K, 5 ps^{-1} (steps of 5 ps and 10 K)<br>-	protein and DNA heavy atoms<br>k_1 = 25 kcal/mol·Å^2	-
2 (150 ps, NPT, 1 fs)	300 K, 1 ps-1<br>1 atm, 100 fs, 50 fs	k_1 = 10 kcal/mol·Å^2	-
3 (250 ps, NPT, 1 fs)	300 K, 1 ps-1<br>1 atm, 100 fs, 50 fs	protein structured regions (linker excluded) and DNA<br>k_1 = 5 kcal/mol·Å^2	-
4 (250 ps, NPT, 1 fs)	300 K, 1 ps-1<br>1 atm, 100 fs, 50 fs	k_1 = 1 kcal/mol·Å^2	-
5 (250 ps, NPT, 1 fs)	300 K, 1 ps-1<br>1 atm, 100 fs, 50 fs	Protein structured regions (linker excluded) and DNA backbone<br>k_1 = 1 kcal/mol · Å^2	Watson Crick base pairs and protein-DNA (sidechain-base) interactions<br>k_2 = 25 kcal/mol · Å^2
6 (250 ps, NPT, 1 fs)	300 K, 1 ps-1<br>1 atm, 100 fs, 50 fs	k_1 = 1 kcal/mol · Å^2	k_2 = 10 kcal/mol · Å^2
7 (250 ps, NPT, 1 fs)	300 K, 1 ps-1<br>1 atm, 100 fs, 50 fs	K_1 = 1 kcal/mol · Å^2	k_2 = 5 kcal/mol · Å^2
8 (250 ps, NPT, 1 fs)	300 K, 1 ps-1<br>1 atm, 100 fs, 50 fs	-	k_2 = 1 kcal/mol · Å^2
9 (250 ps, NPT, 1 fs)	300 K, 1 ps-1<br>1 atm, 100 fs, 50 fs	-	k_2 = 0.5 kcal/mol · Å^2
10 (250 ps, NPT, 1 fs)	300 K, 1 ps-1<br>1 atm, 100 fs, 50 fs	-	k_2 = 0.1 kcal/mol · Å^2
11 (250 ps, NPT, 1 fs)	300 K, 1 ps-1<br>1 atm, 100 fs, 50 fs	-	k_2 = 0.05 kcal/mol · Å^2
12 (250 ps, NPT, 1 fs)	300 K, 1 ps-1<br>1 atm, 100 fs, 50 fs	-	k_2 = 0.01 kcal/mol · Å^2
13 (1 ns, NPT, 1 fs)	300 K, 1 ps-1<br>1 atm, 100 fs, 50 fs	-	-
14 (1.5 ns, NPT, 1.5 fs)	300 K, 1 ps-1<br>1 atm, 200 fs, 100 fs	-	-
15 (5 ns, NPT, 2 fs)	300 K, 1 ps-1<br>1 atm, 200 fs, 100 fs	-	-

for hydrogen bonds, the donor acceptor distance threshold was 3.0

for hydrophobic interactions the distance threshold was 4.0 Å

APPENDIX SUPPLEMENTARY TABLE S4:

SEQUENCES OF OLIGONUCLEOTIDES FOR CLONING AND SITE-DIRECTED MUTAGENESIS

Primer name	Sequence 5'->3'
Oct4-151M_F	GGCGCCAGAAGGGCAAAAGAATGAGTATTGAGTATTCCCAACGAGAAG
Oct4-151M_R	CTTCTCGTTGGGAATACTCAATACTCATTCTTTTGCCCTTCTGGCGCC
Oct4-LinkO6_F	CAAGATCGCGGCGCAGGGCCGGAAGAGAAAGCGAACTAGC
Oct4-LinkO6_R	GGGGCTGCCGCTGGACGAGTCGGCTTCCTCCACCCAC
Oct4-7D,22K_F	CATGAAAGCCCTGCAGGATGAGCTAGAACAGTTTGCCAAGCTGCTGAAGCAGAAGAGGATCAAGTTGGGGTACACCC
Oct4-7D,22K_R	GGGTGTACCCCAACTTGATCCTCTTCTGCTTCAGCAGCTTGGCAAACTGTTCTAGCTCATCCTGCAGGGCTTTCATG
Oct4-21Y,29R_F	GCTGAAGCAGAAGAGGTATACCTTGGGGTACACCCAGGCCCGGGTGGGGCTCACCCTGGGCG
Oct4-21Y,29R_R	CGCCCAGGGTGAGCCCCACCCGGGCCTGGGTGTACCCCAAGGTATACCTCTTCTGCTTCAGC
Oct6-151S_F	GCGGCAGAAGGAGAAGCGCTCAACCCCCGCGGCCGGCGCG
Oct6-151S_R	CGCGCCGGCCGCGGGGGTTGAGCGCTTCTCCTTCTGCCGC
Oct6-7K,22T_F	GATGCTCCCAGCTCCAAGGACCTGGAGCAGTTCGCCAAGCAGTTCAAGCAACGACGCATCACGCTGGGCTTCA
Oct6-7K,22T_R	TGAAGCCCAGCGTGATGCGTCGTTGCTTGAACTGCTTGGCGAACTGCTCCAGGTCCTTGGAGCTGGGAGCATC
Oct6-LinkO4_F	GAGACCCTGGTGCAGGCCCGCAAGCGCAAGAAGCGC
Oct6-LinkO4_R	GCATATCTCCTGAAGGTTCTCATTGTTGTCGGTCTCCTCCAGCCAC
Oct6-21Y,29R_F	CAAGCAACGACGCTACAAGCTGGGCTTCACCCAGGCCCGCGTGGGACTGGC
Oct6-21Y,29R_R	GCCAGTCCCACGCGGGCCTGGGTGAAGCCCAGCTTGTAGCGTCGTTGCTTG

APPENDIX SUPPLEMENTARY TABLE S5:

SEQUENCES OF PRIMERS FOR GENOTYPING OR qRT-PCR

Primer name	Sequence 5'->3'
**pMX-Oct4_F**	GTGTGGTGGTACGGGAAA
**pMX-Oct4_R**	GGTGAGAAGGCGAAGTCT
**pMX-Oct6_F**	CACCACCACACACTGCCCGGCTCTG
**pMX-Oct6_R**	CCCTTTTTCTGGAGACTAAATAAAATC
**pMX-Sox2_F**	GTGTGGTGGTACGGGAAA
**pMX-Sox2_R**	TTCAGCTCCGTCTCCATC
**pMX-Klf4_F**	GTGTGGTGGTACGGGAAA
**pMX-Klf4_R**	CGCGAACGTGGAGAAGGA
**pMX-c-Myc_F**	TGGTACGGGAAATCACAA
**pMX-c-Myc_R**	GTCATAGTTCCTGTTGGT
**Esrrb_F**	AGGCTCTCATTTGGGCCTAGC
**Esrrb_R**	ATCCTTGCCTGCCACCTGTT
**Fgf4_F**	GGGAGGCTACAGACAGCAAG
**Fgf4_R**	CTGTGAGCCACCAGACAGAA
**Gapdh_F**	CCAATGTGTCCGTCGTGGAT
**Gapdh_R**	TGCCTGCTTCACCACCTTCT
**Klf4_F**	TGTGTCGGAGGAAGAGGAAGC
**Klf4_R**	ACGACTCACCAAGCACCATCA
**Nanog_F**	GAACGGCCAGCCTTGGAAT
**Nanog_R**	GCAACTGTACGTAAGGCTGCAGAA
**Sox2_F**	TTCGAGGAAAGGGTTCTTGCTG
**Sox2_R**	TCCTTCCTTGTTTGTAACGGTCCT
**Rex1_F**	GGCTGCGAGAAGAGCTTTATTCA
**Rex1_R**	AGCATTTCTTCCCGGCCTTT
**Utf1_F**	ACGTGGAGCATCTACGAGGT
**Utf1_R**	TAGACTGGGGGTCGTTTCTG
**WPRE_F**	TGTTGCCACCTGGATTCTGC
**WPRE_R**	AGGAAGGTCCGCTGGATTGA

APPENDIX FIGURE LEGENDS

Appendix Figure S1. Two Oct4/Oct6 linker alignments result in a different reprogramming efficiency with the Oct4^{LinkO6} mutant.

A. Two sequence alignments of POU linker regions of Oct4 and Oct6. On the left, first sequence alignment used by Esch and coworkers [44]. On the right, a structural alignment of the Oct4 and Oct6 linker sequences is shown. In the structural alignment, the entire RK motifs are aligned with a central gap (see Appendix Fig. S1B), thus shifting the position of the first Arg residue in the Oct6 RK motif by one position compared to the sequence based alignment. Prominent residues are highlighted by their number in the sequence.

B. Structural model illustrating the difference between Oct4 (green) and Oct6 (orange) RK motifs, which follow the linkers. The "gap" points to the region where Oct4 lacks one positive charge compared with Oct6. This region is also marked by a blue arrow in the structural alignment in panel A of this Figure. Additionally, residue 40 of the POU_S (Lys in Oct4 and Gln in Oct6) is marked with an asterisk. This residue comes close to the RK region after the linker and adds one positive charge to the construct used in Esch and coworkers [44].

C. Comparison of reprogramming efficiencies using WT Oct4 TF and the two Oct4^{LinkO6} mutants—first based on the new structural alignment presented here (excluding the Arg residue) and second using the alignment used by Esch and coworkers (including the Arg residue; n=1).

Appendix Figure S2. Transgene expression and illustration of colony counting.

A. The relative transcript levels of Oct4 and its mutants were analyzed by qRT-PCR. Viral supernatants from reprogramming experiments performed side-by-side are shown as mean +/- standard deviation (n=2).

B. Representative images of GFP-positive colonies (at 2.5x magnification) generated by WT Oct4 and its mutants in combination with Sox2, Klf4, and c-Myc. Colonies were counted 16 days after viral infection, using a fluorescence microscope. GFP signals considered independent colonies are marked with dashed circles. Scale bars: 500 µm.

APPENDIX FIGURES

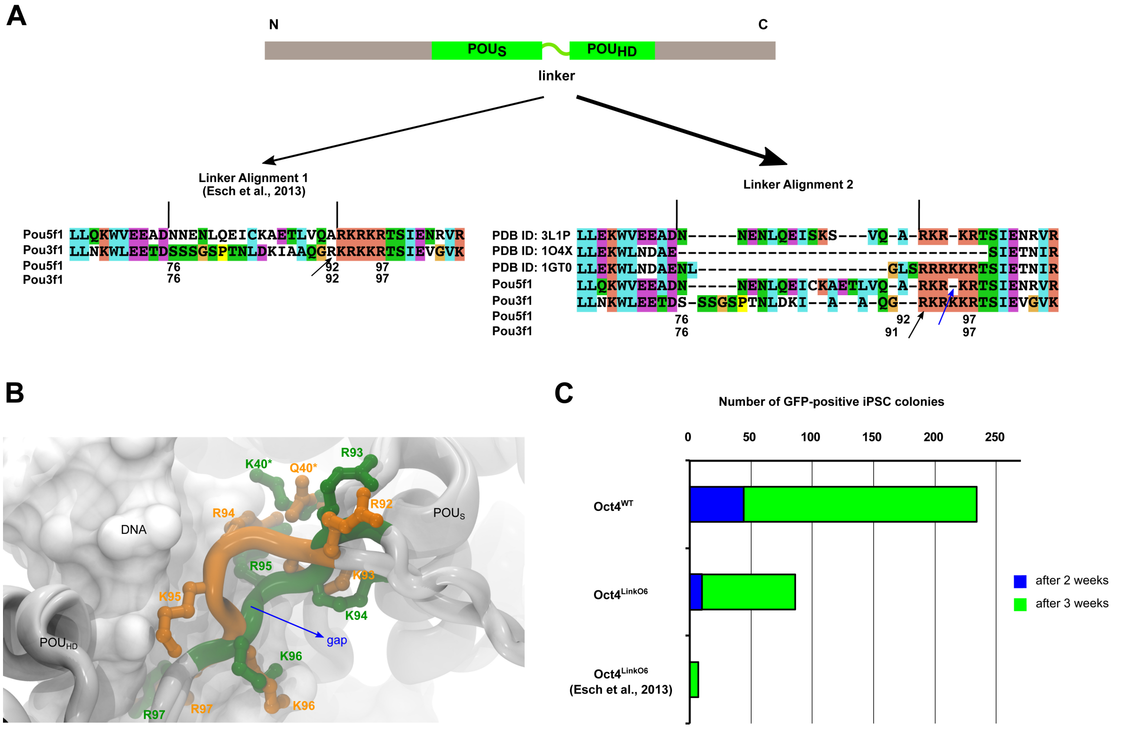

Appendix Figure S1. Two Oct4/Oct6 linker alignments result in a different reprogramming efficiency with the Oct4$^{\text{LinkO6}}$ mutant.

APPENDIX FIGURES

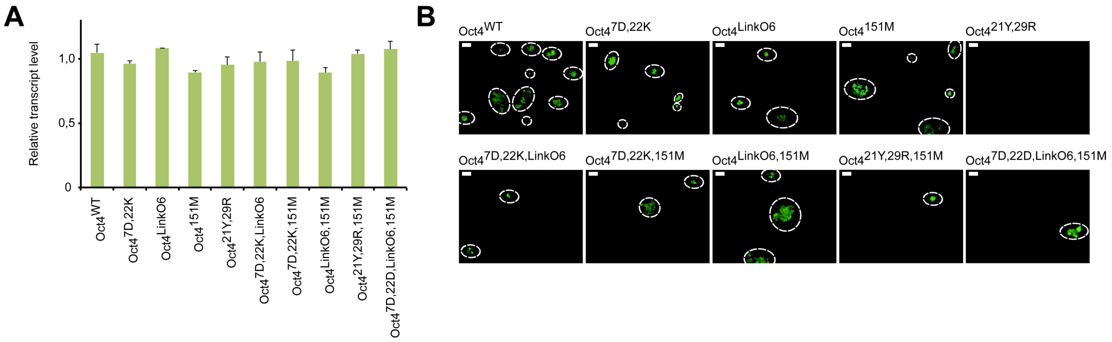

Appendix Figure S2. Transgene expression and illustration of colony counting.
