# Supplementary material for: Changing POU dimerization preferences converts Oct6 into a pluripotency inducer
Source: EMBO Rep. 2016 Dec 22;18(2):319–33. doi: 10.15252/embr.201642958 (PMC5286379; doi:10.15252/embr.201642958)
Supplement: Supplementary file 2 — Expanded View Figures PDF [file EMBR-18-319-s002.pdf]

Expanded View Figures

Figure EV1. The POU factor Oct4 exhibits a unique DNA-binding preference.

A To count the relative abundance of the *OctOct* (*MORE*) versus *SoxOct* motif, the presence of position weight matrices obtained was analyzed using HOMER (lower panel) [39]. References for ChIP-Seq data sets are in order (left to right): Oct2 in B cells [39], Brn2 after 48 h of its overexpression in MEFs [54], Brn2 in NPCs [54], Brn2 in NPCs [55], and Oct4 in ESCs [72]. The upper panel was generated using word searches with IUPAC strings instead of pwms on the same set of ChIPseq peaks as in the lower panel.

B Representative EMSAs used for omega calculations showing the formation of Oct–Oct homodimers on the *MORE* DNA element for a panel of seven POU proteins. Complexes were separated on native gels and subsequently imaged. Note, in Fig 1E and F, only data for Oct1 but not the closely related Oct11 are shown.

C Representative EMSAs performed as single-tube reactions containing the Sox2 HMG and POU domains of Oct transcription factors, in combination with Cy5-labeled *SoxOct* and FAM-labeled *MORE* DNA elements. Complexes were separated on native gels and sequentially imaged using FAM and Cy5 channels.

D Bar plots representing differences in DNA-binding preference as determined by single-tube EMSA reactions for a panel of six POU proteins (classes I-VI) represented as log2 ratios of hetero- and homodimer band intensities on Cy5-*OctSox* and FAM-*MORE* DNA elements, respectively. Individual data points are shown as gray jitter plot. The mean is shown with standard deviation as error bars (n = 9), and Tukey's multiple comparison of means was performed to assess statistical significance (***P < 0.001).

Source data are available online for this figure.

 © 2016 The Authors

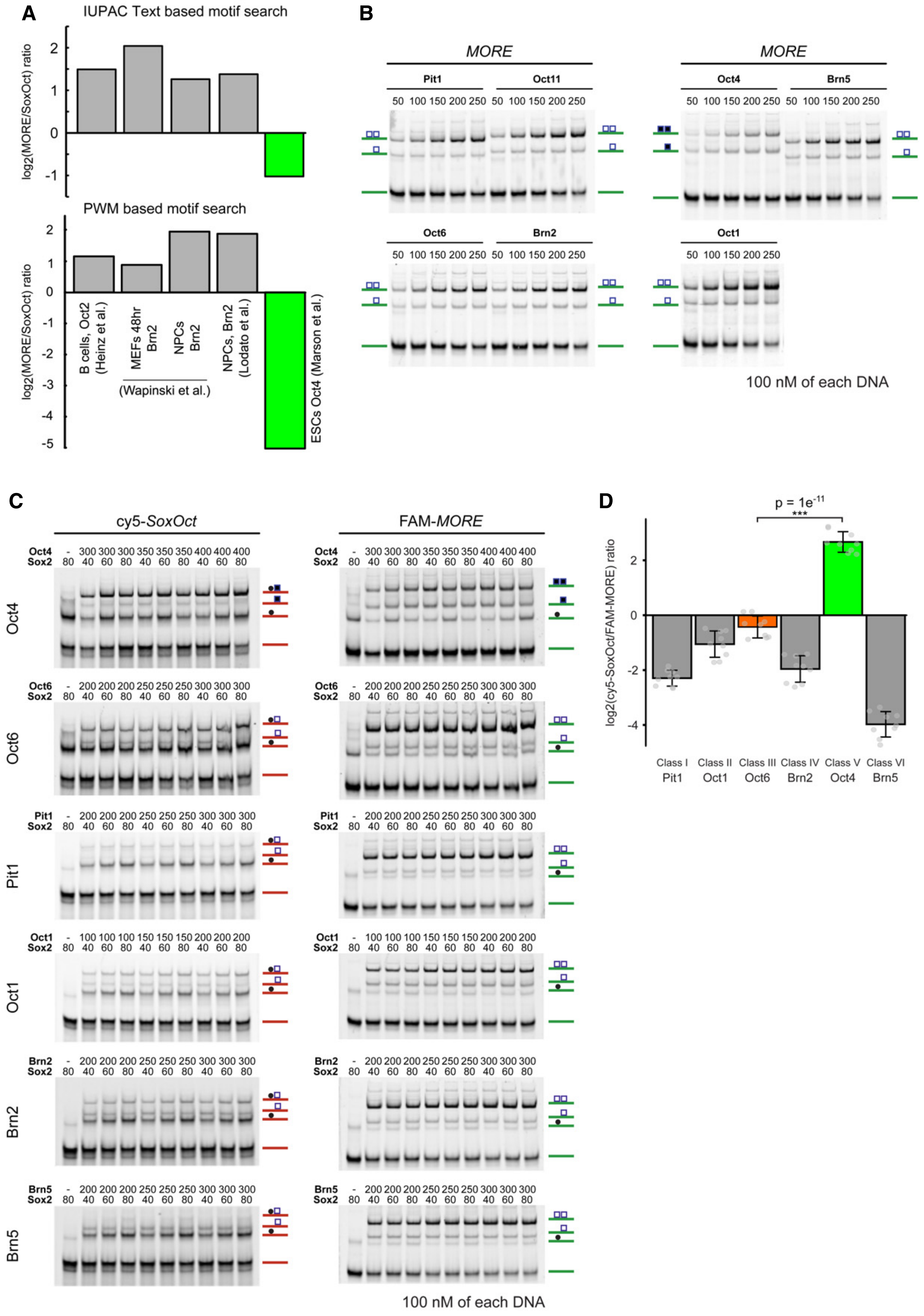
A
IUPAC Text based motif search
log2(MORE/SoxOct) ratio
PWM based motif search
B cells, Oct2 (Heinz et al.)
MEFs 48hr Brn2
NPCs Brn2
NPCs, Brn2 (Lodato et al.)
ESCs Oct4 (Marson et al.)
(Wapinski et al.)
B
MORE
Pit1
Oct11
Oct4
Brn5
Oct6
Brn2
Oct1
50 100 150 200 250
100 nM of each DNA
C
cy5-SoxOct
FAM-MORE
Oct4
Oct6
Pit1
Oct1
Brn2
Brn5
Sox2
100 nM of each DNA
D
p = 1e-11
***
log2(cy5-SoxOct/FAM-MORE) ratio
Class I Pit1
Class II Oct1
Class III Oct6
Class IV Brn2
Class V Oct4
Class VI Brn5

Figure EV1.

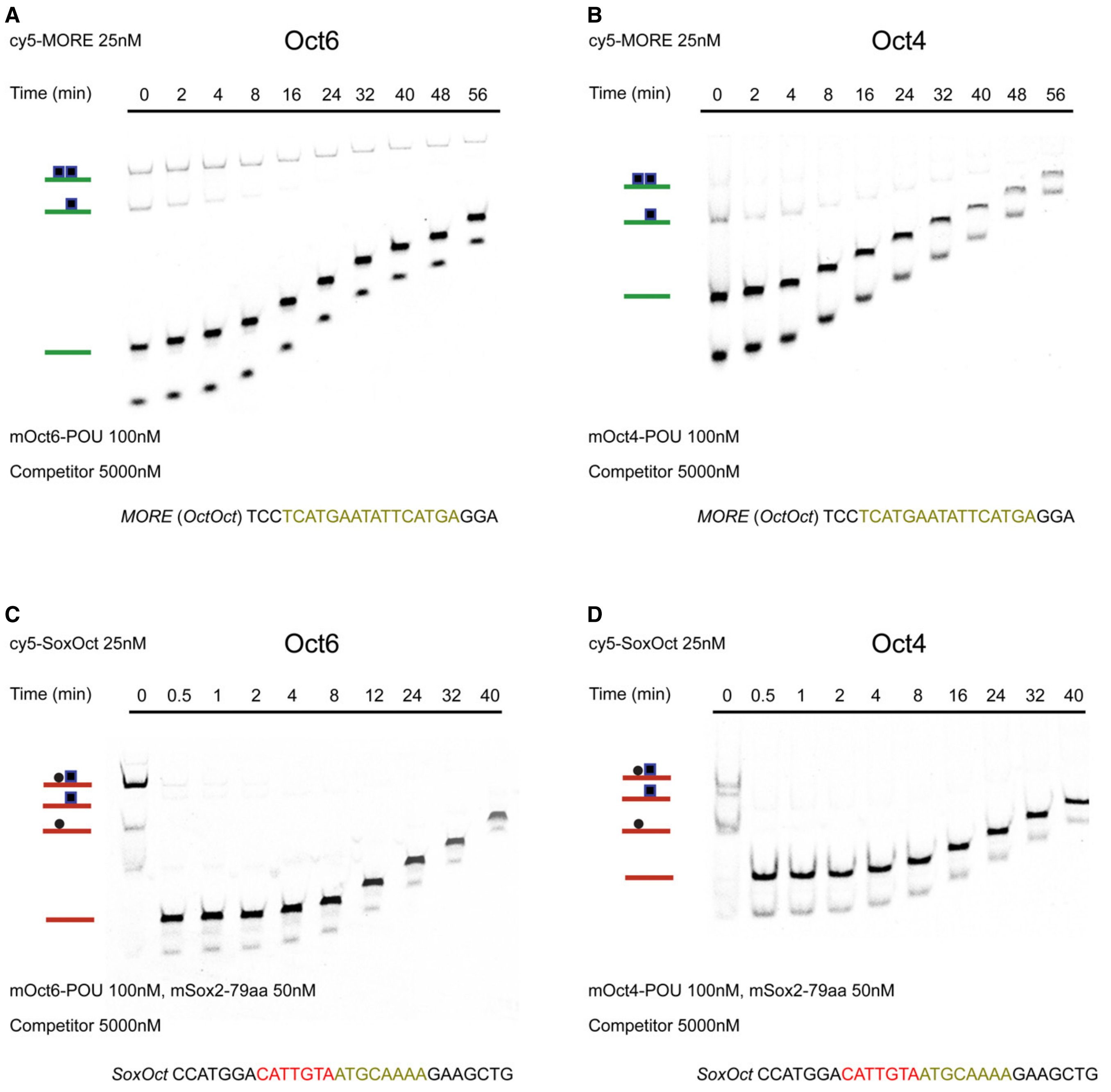

Figure EV2. Oct6 forms a long-lived homodimer on *MORE* DNA.

A–D Off-rate EMSA using the mouse Oct6 POU (A, C) and the mouse Oct4 POU (B, D) along with *MORE* (A, B) or *SoxOct* (C, D) DNA elements, respectively. Dissociation was initiated after incubation of proteins with Cy5-labeled DNA for 1 h by the addition of a 200-fold excess of unlabeled competitor DNA of the same sequences as the Cy5-labeled reporter DNA. Reactions were prepared in single tubes and directly loaded onto running gels after indicated time points. Concentrations of the reactants and sequences of the forward DNA strand are mentioned.

 © 2016 The Authors

Figure EV3. An exchange of Met and Ser residues in Oct4 and Oct6 POU domains is responsible for different DNA-binding preferences.

A Sequence alignment of POU domains of Oct family TFs. POU_S, the linker region, and POU_{HD}, as well as individual helixes, are labeled. Notably, this structure-based alignment differs slightly from the one used previously [44], which is explained by a low conservation of residues in the N-terminal part of the POU linker. The alignment was prepared in T-Coffee software and colors distinguish conservation and amino acid residue types. A protein surface interacting with the *MORE* DNA (as shown in [17]) is highlighted. Each of three studied residues (B) is marked by a black asterisk.

B An overview of tested Oct4 and Oct6 mutants with amino acid exchanges in their POU_{HD}. The EMSA below shows the low cooperativity on *MORE* DNA element caused by the mutations. As the single mutation of the 151 site had the same effect as double and triple mutations, the single mutation was selected for further study.

C Bar plots of EMSA-derived cooperativity factors show different preferences between formation of Oct–Oct homodimers on *MORE* (left) and Sox2–Oct heterodimers on the *SoxOct* element (right) for WT and mutated forms of Oct4 and Oct6 POU domains. Cooperativity values for WT Oct4 and Oct6 correspond to the values shown in Fig 1E and F and are shown again for comparison with the mutant proteins. The mean is shown with standard deviation as error bars (n = 3–13), and Tukey's multiple comparison of means was performed to assess statistical significance (***$P < 0.001$).

D Representative EMSAs performed in single-tube reactions containing the Sox2 HMG and $Oct4^{151M}$ and $Oct6^{151S}$ POU domains of Oct transcription factors. Related to the quantification in Fig 2D.

© 2016 The Authors

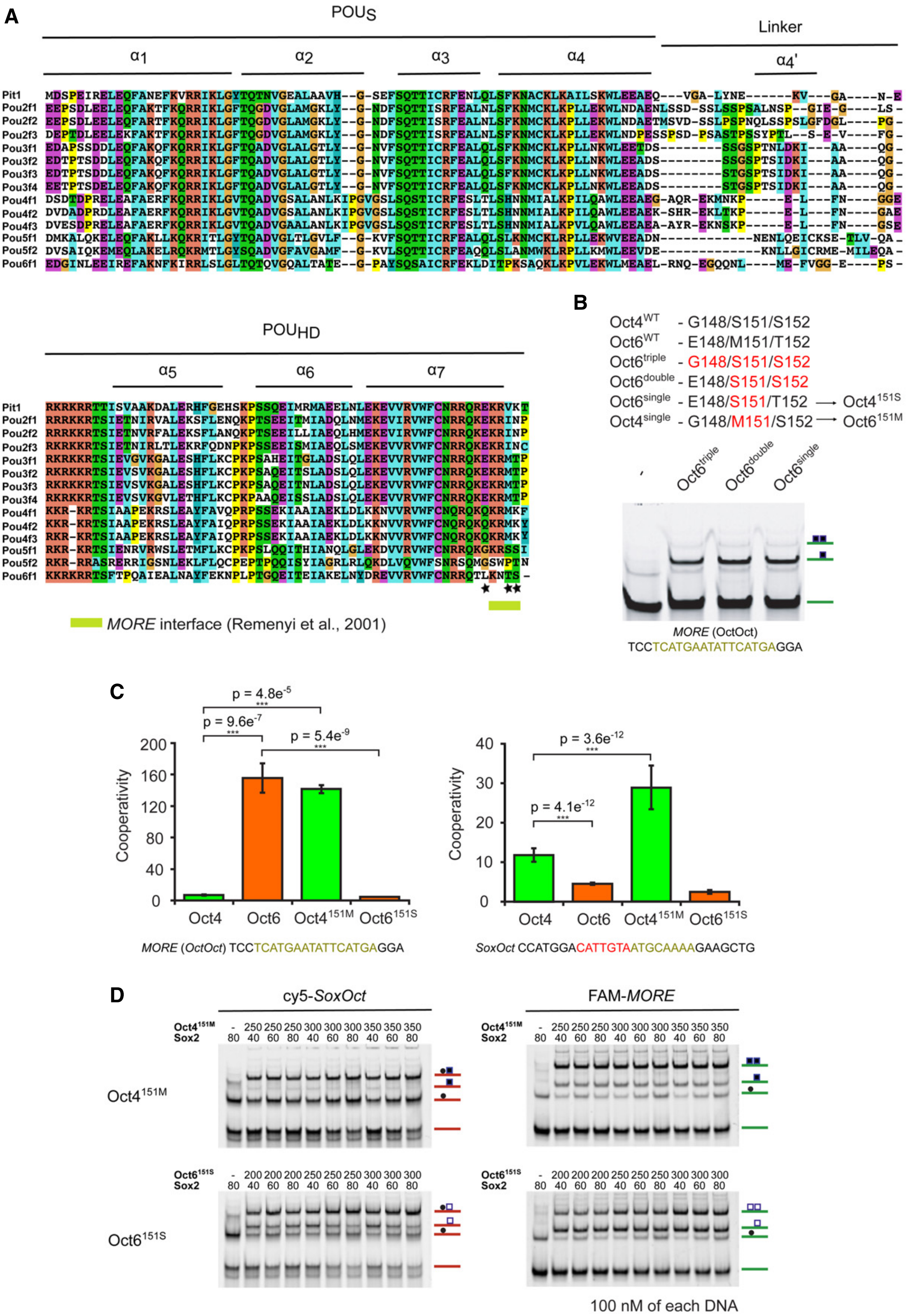

Figure EV3.

 © 2016 The Authors

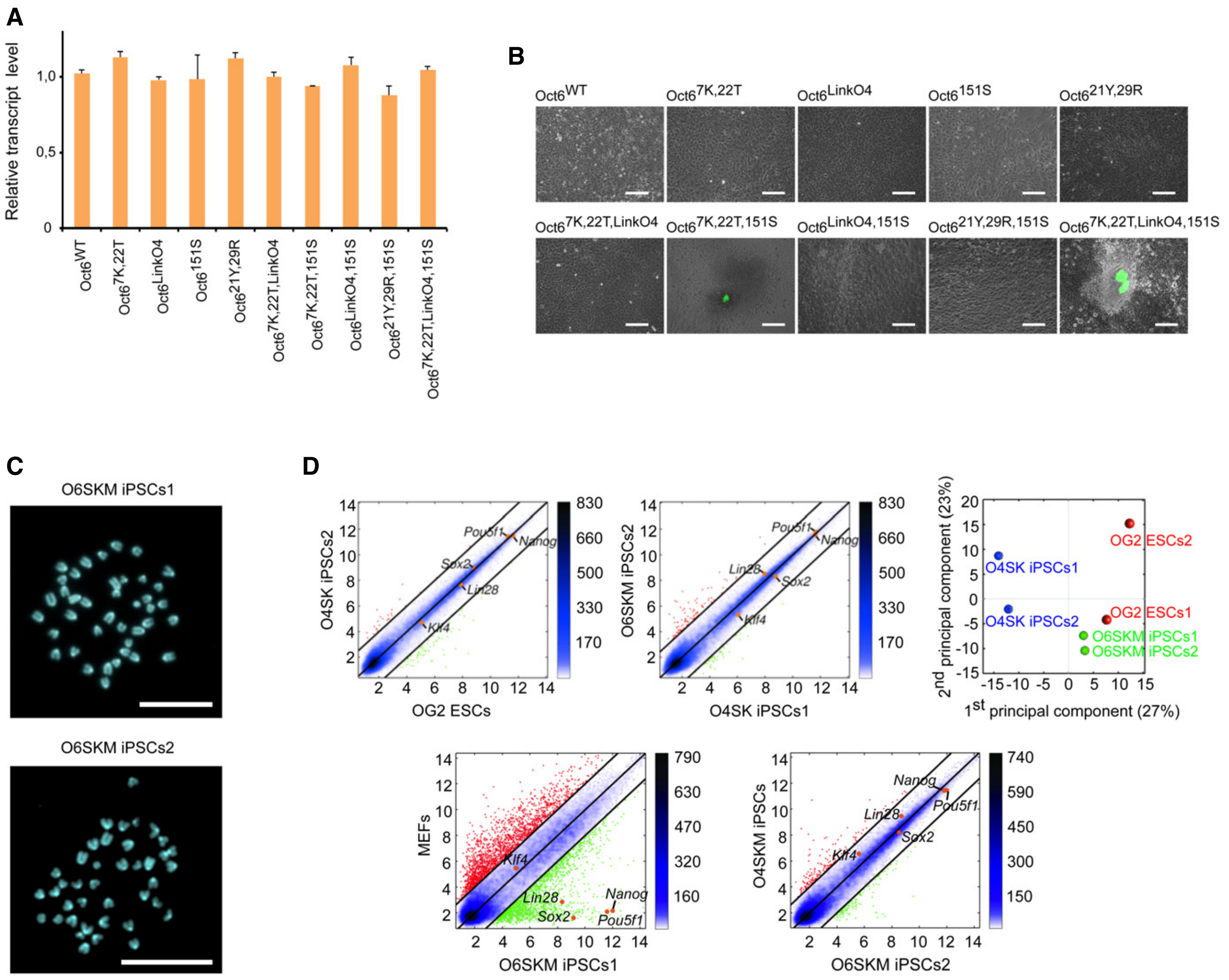

Figure EV4. O6SKM-induced pluripotent cells have normal karyotype and global gene expression profile, similar to embryonic stem cells.

A The relative transcript levels of Oct6 and its mutants were analyzed by qRT–PCR. The mean values for biological replicates using viral supernatants from same reprogramming experiments are shown with the standard deviation as error bars (n = 2).

B Oct4-GFP-positive colonies of mouse iPSCs generated by Oct6 mutants in combination with Sox2, Klf4, and c-Myc. Colonies were imaged 16 days after viral infection, using a fluorescence microscope. Scale bars: 250 μm.

C Karyotyping of two stable mouse O6SKM iPSC lines revealed that cells from both lines carry the correct number of chromosomes—40. Scale bars: 20 μm.

D Pairwise scatter plots comparing global gene expression profiles of O6SKM iPSC lines with OG2 ESCs, O4SK iPSCs, and previously published O4SKM iPSCs and MEFs. Black lines represent a twofold change in gene expression levels between the paired cell lines. On the right side of the plots, color bar indicates scattering density. Red and green dots represent up- and downregulated genes, respectively. Positions of selected pluripotency-related genes are highlighted as orange points. Principal component analysis highlighting the close relationship between global gene expression profiles of O6SKM iPSCs (green circles), OG2 ESCs (red circles), and O4SK iPSCs (blue circles) is shown in the upper right corner.

Figure EV5. Cells reprogrammed using the synthetic Oct6 molecule show pluripotency *in vivo*.

A Sections of teratomas stained 4 weeks after subcutaneous injection of nude mice with iPSCs generated using O6SKM factors. Teratomas contain all three embryonic germ layers: endoderm (epithelium, e), mesoderm (muscle, m), and ectoderm (keratin, k, and neural epithelium with rosettes, n). Scale bars: 100 μm.

B Oct4-GFP-positive germ cells were detected in the male fetal gonads of E13.5 embryos, confirming the germline contribution of O6SKM iPSCs. Scale bars: 100 μm.

C Oct4-GFP-positive germ cells were detected in the gonads of E19.5 pups. Scale bars: 250 μm.

D Representative example of two chimeric mice generated by O6SKM iPSCs. The agouti coat color originated from O6SKM iPSCs. These two chimeras represent mice numbers 13 and 14 (see genotyping results below).

E PCR analysis of DNA isolated from the tails of chimeric mice was performed in order to demonstrate germline contribution by the presence of Oct4-GFP (OG2) and Oct6 viral transgenes. Contamination by Oct4 virus was also excluded by PCR analysis; M stands for DNA marker, ctrl (+) refers to positive iPSC control, and ctrl (−) refers to negative MEF control. Samples 1–14 refer to O6SKM iPSCs2; samples 15–20 refer to O6SKM iPSCs1. Samples labeled with "X" are not relevant to this study.

F Genotyping of Oct4-GFP-positive pups after germline transmission confirmed the presence of Oct6 viral transgene. M stands for DNA marker. Two GFP-negative mice were used as negative controls (ctrl1 and ctrl2).

G The Oct4 complementation assay using ZHBTc4 embryonic stem cells was performed as was described previously [68]. Oct4 and engineered Oct6 transgenes were used to rescue the ES cells upon loss of endogenous Oct4 after the addition of doxycycline (Tc, tetracycline antibiotics) and thus maintain pluripotency. Scale bars: 250 μm.

H The relative transcript levels of selected pluripotency markers and also of relative lentiviral levels of WT Oct4 and engineered Oct6 were analyzed by qRT–PCR. The mean values are shown with the standard deviation as error bars (n = 2).

 © 2016 The Authors

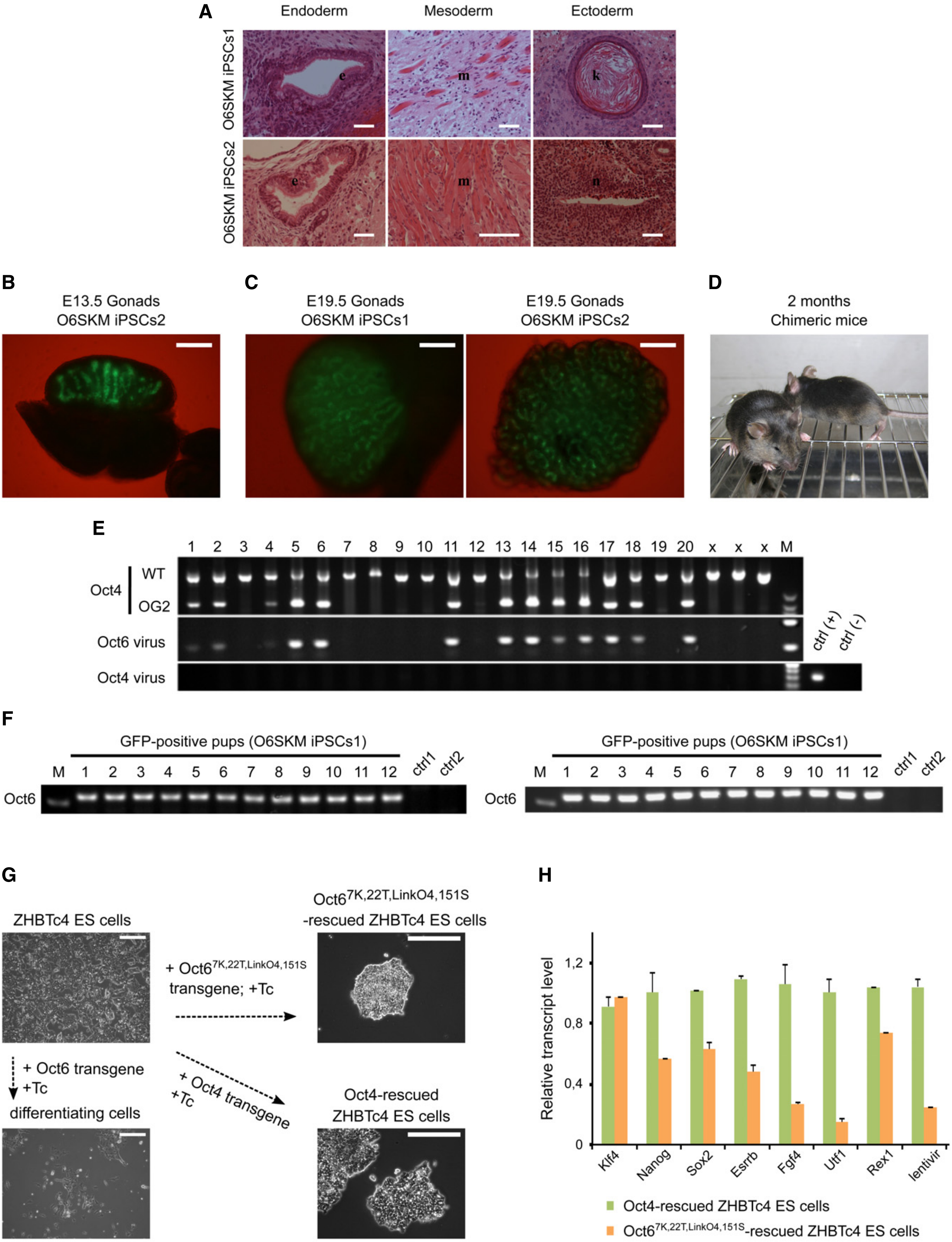
A
Endoderm
Mesoderm
Ectoderm
O6SKM iPSCs1
O6SKM iPSCs2
B
E13.5 Gonads
O6SKM iPSCs2
C
E19.5 Gonads
O6SKM iPSCs1
E19.5 Gonads
O6SKM iPSCs2
D
2 months
Chimeric mice
E
1 2 3 4 5 6 7 8 9 10 11 12 13 14 15 16 17 18 19 20 x x x M
Oct4
WT
OG2
Oct6 virus
Oct4 virus
ctrl (+)
ctrl (-)
F
GFP-positive pups (O6SKM iPSCs1)
M 1 2 3 4 5 6 7 8 9 10 11 12 ctrl1 ctrl2
Oct6
G
ZHBTc4 ES cells
+ Oct6 transgene
+Tc
differentiating cells
+ Oct6^7K,22T,LinkO4,151S transgene; +Tc
Oct6^7K,22T,LinkO4,151S -rescued ZHBTc4 ES cells
+ Oct4 transgene
+Tc
Oct4-rescued ZHBTc4 ES cells
H
Relative transcript level
1,2
0,8
0,4
0
Klf4
Nanog
Sox2
Esrrb
Fgf4
Utf1
Rex1
lentivir
Oct4-rescued ZHBTc4 ES cells
Oct6^7K,22T,LinkO4,151S-rescued ZHBTc4 ES cells

Figure EV5.
